# Avelumab maintenance therapy for node-positive muscle invasive bladder cancer: a report of two cases

**DOI:** 10.3389/fonc.2024.1397738

**Published:** 2024-05-28

**Authors:** Darren M. C. Poon, Lap Yin Ho, Yiu Ming Kwong

**Affiliations:** ^1^ Comprehensive Oncology Centre, Hong Kong Sanatorium & Hospital, The Chinese University of Hong Kong, Hong Kong, Hong Kong SAR, China; ^2^ J ABLE Medical Center, Hong Kong, Hong Kong SAR, China; ^3^ Urology Center, Hong Kong Sanatorium & Hospital, Hong Kong, Hong Kong SAR, China

**Keywords:** urothelial carcinoma, node-positive locally advanced bladder cancer, chemotherapy, chemoradiation therapy, avelumab maintenance therapy, case report

## Abstract

**Background:**

Muscle-invasive bladder cancer (MIBC) with nodal involvement is associated with poor prognosis and high mortality. Treatment of node-positive MIBC is complex due to disease heterogeneity and a lack of evidence-based treatment options, especially alternatives to radical cystectomy. We describe a bladder-sparing management approach involving systemic therapy followed by maintenance therapy, illustrated with two cases of node-positive MIBC.

**Case presentation:**

Two patients with node-positive MIBC received upfront gemcitabine/cisplatin chemotherapy, concurrent chemoradiotherapy (cCRT), and avelumab (immune checkpoint inhibitor) maintenance therapy. Both patients achieved complete remission without recurrence or distant metastasis post-avelumab maintenance therapy. At the last follow-up, Patient 1 (45-year-old male) was in remission for over two years, and Patient 2 (57-year-old male) was in complete remission for over one year post-chemotherapy. Avelumab treatment was well-tolerated, with no immune-related adverse events, and quality of life (QoL) was maintained.

**Conclusion:**

Both cases showed a good response and extended remission on avelumab maintenance, supporting its use in conjunction with local consolidation therapy as a bladder-preserving approach in node-positive MIBC. Further research, such as the ongoing INSPIRE trial, is required to refine treatment strategies for this patient group.

## Introduction

With a global age-adjusted incidence rate of 9.5 per 100,000 for men and 2.4 per 100,000 for women, bladder cancer (BC) is the most common urinary tract malignancy and the tenth most commonly diagnosed cancer (1). In muscle-invasive BC (MIBC), the tumor spreads into or through the muscle layer of the bladder. The prognosis of MIBC is poor, especially when there is metastasis to pelvic lymph nodes (LNs), termed locally advanced node-positive MIBC. This form is graded as at least stage III disease and carries a high risk of progression and mortality (2). Patients with node-positive BC have five-year disease-specific survival rates that are less than half those of patients without nodal involvement (31.2% vs. 66.7%) (3). Historically, patients with clinically node-positive BC have been treated similarly to those with distant metastases. However, recent studies reveal that this population is highly heterogeneous, and there is a lack of evidence-based guidance for their treatment, primarily due to their exclusion or underrepresentation in clinical trials (4).

Node-positive BC is widely regarded as a systemic disease with the probable presence of distant micro-metastases. As such, the management of clinically node-positive disease should ideally be multimodal, including a combination of systemic therapy, surgery, and/or radiation (4). According to international guidelines, the standard treatment for patients with node-positive MIBC includes radical cystectomy (RC) and pelvic lymph node dissection (PLND) before or after chemotherapy in selected patients (5–8). In the National Comprehensive Cancer Network (NCCN) guidelines, the primary treatment options for patients with radiologically suspicious node-positive (cN1) disease, classified as stage IIIA (9), include neoadjuvant chemotherapy (NAC) followed by RC and PLND for those eligible for cisplatin, or surgery alone for patients unfit for cisplatin-based chemotherapy (5). However, RC and PLND are associated with notable morbidity and high mortality risk and have a major impact on quality of life (QoL) (10). An alternative primary treatment approach, bladder preservation, involves maximal transurethral resection of the bladder tumor (TURBT) followed by concurrent chemoradiotherapy (cCRT). This bladder-sparing method is typically reserved for patients with smaller solitary tumors, absence of extensive or multifocal carcinoma *in situ* (CIS), no tumor-related hydronephrosis, and satisfactory pre-treatment bladder function (5–8). In contrast, the primary treatment for stage IIIB (cT1–T4a, N2–3) disease (9) includes either upfront systemic chemotherapy (with consideration of consolidative local therapy) or cCRT (5). The choice between these options should consider the patient’s clinical condition, surgeon and center experience, and patient preferences.

The goal of maintenance therapy is to extend the benefits achieved with first-line systemic therapy and prolong progression-free survival (PFS) and overall survival (OS). In the immunotherapy era, programmed death-ligand 1 (PD-L1) pathway targeting agent avelumab has shown promise as a “switch” maintenance therapy (11, 12). Starting avelumab maintenance therapy within 10 weeks of completing chemotherapy, irrespective of PD-L1 expression status, was shown to prolong PFS and OS in the Phase III JAVELIN Bladder 100 trial (12). In this trial, patients with unresectable locally advanced or metastatic urothelial carcinoma (UC) and no disease progression on first-line platinum-based chemotherapy received avelumab as first-line maintenance treatment along with best supportive care (BSC) (12). Avelumab plus BSC resulted in significantly higher one-year survival rates (58.4% vs. 71.3%) and longer median PFS (2 months vs. 3.7 months) than BSC, leading to accelerated Food and Drug Administration approval of avelumab as a first-line maintenance treatment in locally advanced or metastatic UC. Although the JAVELIN trial reported results for patients with non-visceral disease, it did not specifically analyze the subgroup of patients with clinically node-positive, non-metastatic disease. Therefore, the potential utility of avelumab maintenance therapy in this group remains unclear.

The cases presented here illustrate a bladder-sparing approach for node-positive MIBC, with upfront cisplatin-based chemotherapy and cCRT, followed by avelumab as maintenance therapy.

## Case presentation

### Patient 1

**Figure 1:**
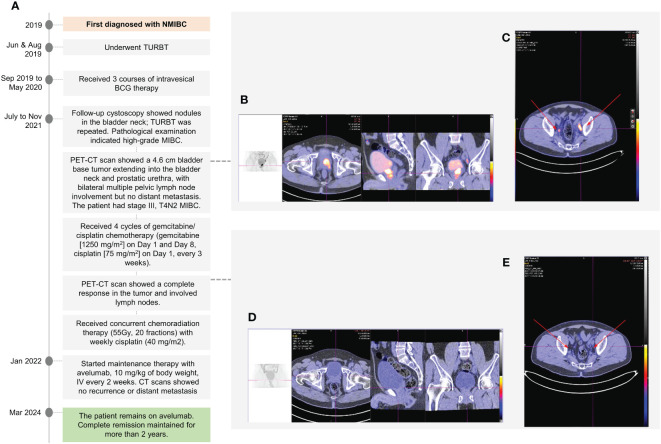
A chronological overview of the key events in the treatment journey of a 45-year-old male with muscle-invasive bladder cancer **(A)**. PET/CT scans showed a 4.6 cm bladder base tumor extending into the bladder neck and prostatic urethra **(B)**, with bilateral multiple pelvic lymph node involvement **(C)**. The patient showed a complete response after 4 cycles of gemcitabine/cisplatin, with disappearance of the tumor **(D)**; all pelvic lymph nodes subsided **(E)**.

A 45-year-old Chinese male, married with one son, non-smoker, and non-drinker, and with no significant past medical history, initially presented in 2019 with non-muscle invasive BC (NMIBC) (**Figure 1A**). TURBT was performed in June and August 2019, followed by three courses of intravesical Bacillus Calmette Guérin (BCG) therapy (September 2019 to May 2020). In July 2021, follow-up cystoscopy showed nodules in the bladder neck, and TURBT was repeated. Nodule biopsy and pathological examination indicated high-grade MIBC. Given the suspicion of nodal involvement or distant metastases, a position-emission tomography-computed tomography (PET-CT) scan was conducted. There was a 4.6 cm bladder base tumor extending into the bladder neck and prostatic urethra with bilateral multiple pelvic LN involvement. No evidence of distant metastasis was found (**Figures 1B, C**). Based on the findings, the patient had stage III, T4N2 MIBC.

After extensive multidisciplinary discussions and consultations regarding treatment, the patient opted against the surgical approach. Consequently, the patient received four cycles of gemcitabine/cisplatin chemotherapy from the end of July to October 2021 (gemcitabine [1250 mg/m²] administered on Day 1 and Day 8, cisplatin [75 mg/m²] on Day 1, every 3 weeks). Post-chemotherapy PET-CT scans revealed a complete response in the tumor and involved LNs (**Figures 1D, E**). This was followed by cCRT (55Gy in 20 fractions) with weekly cisplatin (40mg/m2), which was completed in November 2021.

In January 2022, the patient started maintenance therapy with avelumab [BAVENCIO, Merck KGaA, Darmstadt, Germany] 10 mg/kg of body weight, administered intravenously every 2 weeks. Subsequent follow-up CT scans and the latest PET-CT scan in March 2024 have consistently shown no recurrence or distant metastasis. Additionally, a cystoscopy performed in August 2023 confirmed ongoing complete remission. The patient has now been on avelumab therapy for over two years and remains in complete remission. During treatment, the patient experienced self-limiting Grade 1 skin itchiness and fatigue, which did not impact daily activities. No immune-related adverse events were observed, and the patient’s QoL was maintained.

### Patient 2

**Figure 2:**
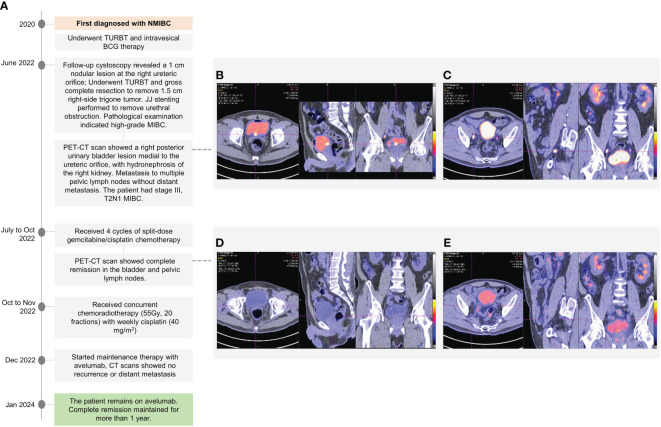
A chronological overview of the key events in the treatment journey of a 57-year-old male with muscle-invasive bladder cancer **(A)**. PET/CT scans showed a right posterior urinary bladder lesion medial to the ureteral orifice **(B)** with associated right hydronephrosis and involvement of multiple pelvic lymph nodes **(C)**. The patient showed a good response after 4 cycles of gemcitabine/cisplatin, with complete remission in the bladder and pelvic lymph nodes **(D, E)**, along with resolution of the hydronephrosis.

The second patient, a 57-year-old male, non-smoker and non-drinker who tested negative for hepatitis B virus surface antigen and was allergic to augmentin, was diagnosed with NMIBC in 2020. His past medical history included hypertension, diabetes mellitus, hyperlipidemia, and depression. His initial management involved TURBT and intravesical BCG therapy (**Figure 2A**). In June 2022, follow-up cystoscopy revealed the presence of a 1 cm nodular lesion at the right ureteric orifice. TURBT followed by gross complete resection was performed to remove a 1.5 cm right-side trigone tumor compressing the ureteric orifice, and JJ stenting was performed to deal with the ureteral obstruction. The pathological findings indicated high-grade MIBC. A PET-CT scan revealed a right posterior urinary bladder lesion medial to the ureteric orifice, with associated hydronephrosis of the right kidney. Metastasis to multiple pelvic lymph nodes was noted but without distant metastasis (**Figures 2B, C**). Based on the findings, the patient had stage III, T2N1 MIBC. Serum creatinine levels were markedly elevated (140 μmol/L; calculated CrCl of 51mL/min), and measured creatinine clearance in a 24-hour urine collection was 72mL/min.

Following multidisciplinary discussions and consultations, the patient chose a non-surgical management approach. In July 2022, the patient started four cycles of split-dose gemcitabine/cisplatin chemotherapy, which was completed in early October 2022. A post-chemotherapy PET-CT scan showed complete remission in the bladder and pelvic LNs (**Figures 2D, E**). The hydronephrosis was resolved, and the JJ stent was removed. The patient then received cCRT (55Gy, 20 fractions) with weekly cisplatin (40mg/m2). In December 2022, 3 weeks after completing cCRT, the patient started maintenance therapy with avelumab. A follow-up PET-CT scan in January 2024 showed complete remission with no recurrence. Additionally, a follow-up cystoscopy is scheduled for mid-2024 to continue monitoring. The patient has now been on avelumab therapy for over a year and remains in complete remission. During treatment, the patient experienced self-limiting Grade 1 skin itchiness and fatigue, which did not impact daily activities, and the patient’s QoL was maintained.

## Discussion and conclusion

Until recently, node-positive MIBC was categorized as stage IV disease irrespective of the extent of nodal involvement and was often excluded from key clinical trials, limiting treatment guidance to small, observational studies (4). Now, it has been accepted that clinically regional node-positive BC is a heterogeneous entity, with some notable differences in prognosis and management approach depending on the extent of nodal involvement (4, 13). There is a general agreement that managing clinically node-positive disease should involve a multimodal approach, incorporating systemic therapy, surgery, and/or radiation. Current guidelines indicate that for patients with stage IIIA MIBC, the general approach includes RC coupled with NAC for those eligible for cisplatin. Alternatively, for patients suitable for a bladder-sparing approach, bladder preservation involving maximal TURBT followed by cCRT is an NCCN category 1 recommended option since 2022 (13). For stage IIIB MIBC patients, treatment typically involves either upfront systemic chemotherapy (with consideration of consolidative local therapy) or cCRT (5). Additionally, the use of avelumab as a “switch” maintenance therapy can be offered to patients with unresectable node-positive BC who achieved at least stable disease following initial chemotherapy (12, 14).

The two cases presented here illustrate the use of avelumab maintenance therapy as part of a bladder-preserving multimodal approach for patients with clinically regional node-positive, non-metastatic MIBC. At the time MIBC was discovered, both patients had clinically node-positive disease, albeit limited to multiple pelvic LNs without visceral or distant metastasis. Following multidisciplinary discussions and consultation with the patients, both patients expressed a preference for non-surgical management. Consequently, a bladder-preserving approach was pursued in both cases: upfront gemcitabine/cisplatin followed by potential consolidative cCRT based on the chemotherapy response, with the option for avelumab maintenance therapy thereafter. Both patients showed excellent responses after four cycles of gemcitabine/cisplatin, with their post-chemotherapy PET-CT scans showing remission of metabolic activity in bladder lesions and pelvic LNs, along with substantial shrinkage or disappearance of the tumors. Patient 1 started avelumab approximately 4 weeks after completing cCRT, and Patient 2 began treatment at around 3 weeks post-cCRT. Both timeframes were within the 10-week window indicated by the JAVELIN trial findings. Avelumab treatment (over two years for patient 1 and one year for patient 2) was well-tolerated by both patients, with no immune-related adverse events, and QoL was maintained. The most recent follow-up scans showed both patients remaining in a state of complete remission at 29 months and 16 months after chemotherapy, respectively, with no disease recurrence.

Close active surveillance after upfront systemic therapy is key for early detection of recurrence and evaluation of subsequent therapy options. Considering the presence of regional node-positive disease in both cases, we reasoned that following chemotherapy with consolidation cCRT would offer the best chance of local and systemic disease control. Moreover, analyses suggest a limited benefit of RC with PLND in patients with node-positive disease (15), which must be weighed against its notable negative impact on QoL and morbidity.

Finally, we explored the use of avelumab maintenance therapy, already a recommended post-chemotherapy option (5), in the context of consolidative cCRT. These two cases suggest that post-chemotherapy maintenance of remission with avelumab is still possible with the interposition of cCRT and that the resulting delay did not compromise disease control. Similarly, there has been an interest in establishing whether consolidative radiotherapy can provide additional benefits in the context of avelumab maintenance therapy (16).

The cases discussed here corroborate recent analyses that underscore the benefits of maintenance therapy in MIBC treatment paradigms, namely, prolonged survival without compromising QoL (11, 17). Data from the ongoing INSPIRE trial (the first prospective study dedicated to addressing node-positive MIBC) will provide insight into both short-term and long-term outcomes for bladder-preserving treatments in this patient population and has the potential to define new treatment strategies for stage III MIBC.

Observations from these cases provide support for extending the immune checkpoint inhibitor maintenance paradigm to encompass local consolidation therapy, which is potentially valuable for defining future bladder-preserving strategies in MIBC.

## Data availability statement

The original contributions presented in the study are included in the article/supplementary material. Further inquiries can be directed to the corresponding author.

## Ethics statement

Written informed consent was obtained from the individual(s) for the publication of any potentially identifiable images or data included in this article.

## Author contributions

DP: Writing – review & editing, Conceptualization, Data curation. LH: Writing – review & editing, Data curation. YK: Writing – review & editing, Data curation.
